# Astrocytes and Microglia Are Resistant to NAD^+^-Mediated Cell Death Along the ARTC2/P2X7 Axis

**DOI:** 10.3389/fnmol.2019.00330

**Published:** 2020-01-14

**Authors:** Björn Rissiek, Joschi Stabernack, Maike Cordes, Yinghui Duan, Sarah Behr, Stephan Menzel, Tim Magnus, Friedrich Koch-Nolte

**Affiliations:** ^1^Department of Neurology, University Medical Centre Hamburg-Eppendorf, Hamburg, Germany; ^2^Institute of Immunology at University Medical Centre Hamburg-Eppendorf, Hamburg, Germany

**Keywords:** P2X7, ARTC2, NAD^+^, ADP-ribosylation, astrocytes, microglia

## Abstract

ADP-ribosylation of the P2X7k splice variant on mouse T cells by Ecto-ADP-ribosyltransferase ARTC2.2 in response to its substrate extracellular nicotinamide adenine dinucleotide (NAD^+^) triggers cell death. Since NAD^+^ is released as a danger signal during tissue damage, this NAD^+^-induced cell death (NICD) may impact the survival of other cell populations co-expressing P2X7 and of one of the ARTC2 isoforms (ARTC2.1, ARTC2.2). NICD of brain-resident, non-T cell populations has only been rudimentarily investigated. In this study, we evaluated the susceptibility of two glia cell populations, astrocytes and microglia, towards NICD. We found that astrocytes and microglia strongly upregulate cell surface levels of ARTC2.1 and ADP-ribosylation of cell surface proteins in response to treatment with lipopolysaccharide (LPS) and the mitogen-activated protein kinase kinase (MEK) 1 and 2 inhibitor U0126, but do not respond to extracellular NAD^+^ with P2X7 activation and induction of cell death. Furthermore, we found that astrocytes and microglia preferentially express the ADP-ribosylation-insensitive P2X7a splice variant, likely accounting for the resistance of these cells to NICD.

## Introduction

P2X receptors are a family of ion channels that are gated by extracellular adenosine triphosphate (ATP). A well-characterized member of this protein family is P2X7, which plays an important role in several immunological processes such as inflammasome formation, release of leaderless pro-inflammatory cytokines, mitochondrial metabolism and cell death (Ferrari et al., 1997; Idzko et al., 2014; Borges da Silva et al., 2018; Linden et al., 2019). In mice, an alternative way of P2X7 activation exists, triggered by a post-translational modification of P2X7 catalyzed by cell surface ADP-ribosyltransferases (ecto-ARTCs). ARTCs use extracellular nicotinamide adenine dinucleotide (NAD^+^) as a substrate to ADP-ribosylate various cell surface proteins, including P2X7 (Seman et al., 2003). The predominant ecto-ARTC expressed by murine immune cells is ARTC2 with its two isoforms ARTC2.1 and ARTC2.2 (Koch-Nolte et al., 1996). While ARTC2.1 is mainly expressed by cells of the innate immune system such as macrophages and microglia, ARTC2.2 is mainly found on T cells. Of note, the enzymatic activity of ARTC2.1 is enhanced in the presence of reducing agents such as dithiothreitol (DTT), which are thought to break a disulfide bond near the protein surface that is unique to ARTC2.1, between Cys-80 and Cys-201 (Hara et al., 1999; Hong et al., 2007). Further, it has been demonstrated that both isoforms are able to ADP-ribosylate P2X7 (Hong et al., 2009b). However, the consequence of P2X7 ADP-ribosylation differs among immune cell populations: ADP-ribosylation of P2X7 on T cells induces gating of P2X7, calcium influx, shedding of cell surface proteins, externalization of phosphatidylserine and ultimately cell death (Seman et al., 2003; Rissiek et al., 2015). This can also be induced *via* ATP-mediated P2X7 activation, however, 10-fold lower NAD^+^ concentrations (30 μM) are sufficient to induce effects comparable to that of ATP (300 μM). This makes extracellular NAD^+^ a potent regulator of T cell death. For macrophages it has been reported that ADP-ribosylation of P2X7 does not induce P2X7 gating, however, it increases the sensitivity of P2X7 towards ATP, thereby lowering the threshold for ATP to induce channel gating (Hong et al., 2009b). Nevertheless, P2X7-mediated induction of cell death can also be achieved in macrophages by prolonged incubation in the presence of ATP. This differential reaction of P2X7 on T cells and macrophages towards ADP-ribosylation has been explained by the expression of two different P2X7 splice variants in macrophages and T cells. While macrophages express the P2X7a variant, T cells express an alternative P2X7 splice variant, termed P2X7k, that differs from the P2X7a in the N-terminal 42 amino acid residues composing the first cytosolic domain and most of the first transmembrane domain (Nicke et al., 2009). Comparative analyses of P2X7a and P2X7k revealed that only the T cell P2X7k variant is gated by ADP-ribosylation, thereby explaining the lack of reactivity of P2X7 on macrophages towards extracellular NAD^+^ (Schwarz et al., 2012).

While the role of ARTC2-mediated ADP-ribosylation of P2X7 has been studied extensively in T cell biology and also in the context of macrophages, not much is known about the impact of this post-translational P2X7 modification on other cell populations. Microglia and astrocytes are two glial cell populations in the brain with important functions in e.g., immune surveillance and neuronal nutrition. Our own recent results point towards a potential ADP-ribosylation of P2X7 on microglia (Rissiek et al., 2017). Further, is has been suggested that NAD^+^ can also trigger cell death along the ARTC2/P2X7 axis in astrocytes (Wang et al., 2012). The ubiquity of NAD^+^ in every metabolically active cell has the consequence that it can be released, analogously to ATP, as danger signal during tissue damage e.g., after ischemic stroke in the brain. Therefore, it is important to know, if the released NAD^+^ has an impact on the vitality of microglia and astrocytes in an ARTC2/P2X7-dependent fashion. In the present study, we evaluated this on astrocytes and microglia from mouse mixed glial cultures.

## Materials and Methods

### Mice

C57BL/6 WT, Balb/c WT, Balb/c ARTC2.1ko (Ohlrogge et al., 2002) and NZW WT mice were bred at the animal facility of the University Medical Center (UKE). ICR mice were purchased from Charles River, Sulzfeld, Germany. All experiments involving tissue derived from animals were performed with the approval of the responsible regulatory committee (Hamburger Behörde für Gesundheit und Verbraucherschutz, Veterinärwesen/Lebensmittelsicherheit, ORG-722). All methods were performed in accordance with the relevant guidelines and regulations.

### Isolation of Primary Brain Microglia, Peritoneal Macrophages, and Spleen T Cells

For the isolation of brain microglia, mice were sacrificed and single-cell suspensions were prepared by collagenase digestion at 37°C for 30 min. The generated cell suspension was filtered through a 70 μm cell strainer and centrifuged for 5 min at 300 *g*. Microglia were separated from debris by resuspending the pellet in 5 ml 33% percoll solution (GE Healthcare, Chicago, IL, USA). The supernatant was removed and the pellet was resuspended in 1 ml ACK erythrocyte lysis buffer and incubated for 1 min on ice to remove erythrocytes. Cells were washed with 10 ml FACS buffer (PBS + 0.2% BSA/1 mM EDTA) and resuspended in FACS buffer. For the isolation of peritoneal macrophages, mice were sacrificed, 5 ml PBS + 1 mM EDTA were injected into the peritoneal cavity to collect peritoneal macrophages by lavage. For the isolation of spleen T cells, mice were sacrificed, the spleen was collected and minced through a 70 μm cell strainer using a syringe piston. The cell suspension was centrifuged for 5 min at 300 *g*, erythrocytes were removed as described above and the cells were finally resuspended in FACS buffer.

### Mixed Glial Cell Cultures and Stimulation With LPS/U0126

Brains from 1 to 2 days old neonatal mice were prepared and transferred into Hanks Balanced Salt Solution (HBSS, Thermo Fisher Scientific, Waltham, MA, USA) containing 10 mM HEPES (Thermo Fisher Scientific, Waltham, MA, USA). After removal of the meninges, brains were minced into smaller pieces, washed and incubated for 25 min in HBSS + 10 mM HEPES with 0.5 mg/ml papain (Sigma-Aldrich, St. Louis, MO, USA) and 10 μg/ml DNAse (Roche Diagnostics, Basel, Switzerland). Cells were then washed in BME medium (Life Technologies, Carlsbad, CA, USA), dissociated and then plated at a density of 3 × 10^5^ cells/ml and cultured in BME media supplemented with 10% FCS and 100U/ml penicillin/100 μg/ml streptomycin (Thermo Fisher Scientific, Waltham, MA, USA). Cultures were used for analyses after 14–21 days and contained 20–30% microglia and 60%–70% astrocytes. To induce ecto-ART activity cells were stimulated with LPS (0.1 μg/ml, Sigma-Aldrich, St. Louis, MO, USA) and U0126 (10 μM, Sigma-Aldrich, St. Louis, MO, USA) in culture medium for 24 h at 37°C.

### Antibodies and Flow Cytometry

Cells were analyzed using BD FACSCanto II following staining with fluorochrome-conjugated mAbs: anti-ARTC2.1 (clone R18A136#2; UKE), anti-etheno-ADP-ribose (clone 1G4, UKE; Young and Santella, 1988), anti-CD11b (clone M1/70; BioLegend, San Diego, CA, USA), anti-GLAST (clone ACSA-1; Miltenyi), anti-P2X7 (clones Hano43 and Hano44, UKE), anti-CD45 (30-F11, Biolegend) and anti-CD4 (clone RM4–5; BioLegend, San Diego, CA, USA). Cells were stained and washed in FACS buffer containing PBS + 0.1% BSA + 1 mM EDTA. For flow cytometric analyses microglia were identified as CD11b^+^GLAST^−^ cells and astrocytes as CD11b^−^GLAST^+^.

### Calcium Influx Assay

**Figure 1:**
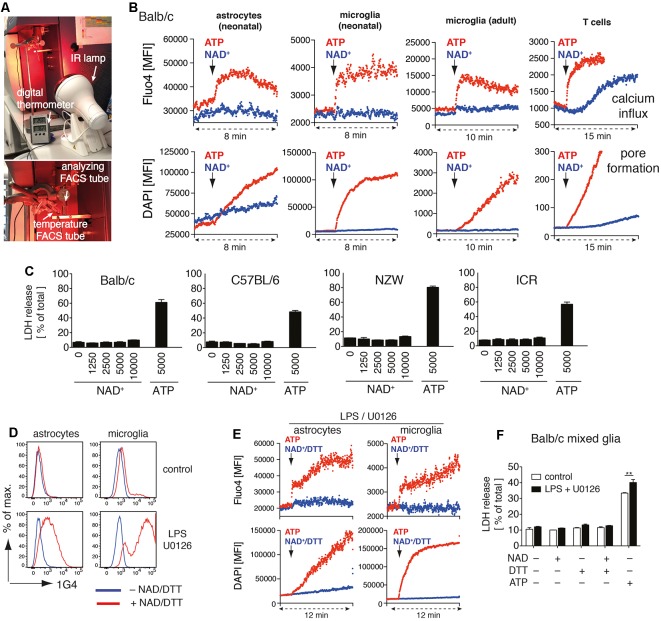
Adenosine triphosphate (ATP) but not nicotinamideadenine dinucleotide (NAD^+^) induces activation of P2X7 on astrocytes and microglia. **(A)** Instrumental setup for 37°C real-time flow cytometry. An IR lamp was placed in close distance to the “analyzing FACS tube” to maintain the sample temperature at 37°C. Temperature was monitored using a digital thermometer in a separate “temperature FACS tube” placed next to the “analyzing FACS tube.” **(B)** Astrocytes and microglia from Balb/c mixed glial cultures, primary adult brain microglia, and splenic CD4 T cells were loaded with Fluo4 and resuspended in PBS supplemented with calcium and DAPI. Calcium influx and pore formation were measured by real-time flow cytometry in response to the addition of ATP (1 mM) or NAD^+^ (1 mM; black arrow) after 2 min of baseline measuring. **(C)** Cell death of mixed glial cells from Balb/c, C57BL/6, NZW and ICR mice in response to NAD^+^ or ATP treatment for 24 h was quantified by measuring LDH release. **(D)** Ecto-ART activity of astrocytes (GLAST^+^CD11b^−^) and microglia (GLAST^−^CD11b^+^) from Balb/c mixed glial cultures (stimulated or not with lipopolysaccharide (LPS)/U0126 for 24 h) was analyzed by flow cytometry following incubation of cells with etheno-NAD^+^ and detection of incorporated etheno-ADP-ribose with etheno-adenosine-specific mAb 1G4. **(E)** Calcium influx and pore formation by LPS/U0126 stimulated cells were measured by real-time flow cytometry as in **(A)**. **(F)** Cell death of LPS/U0126 treated mixed glial cells from Balb/c mice in response to treatment with NAD^+^, NAD^+^/DTT or ATP for 24 h was quantified by measuring LDH release. Statistical comparison of two groups was performed by using the student’s *t*-test (**p < 0.01). Data represent results from two **(B,E,F)** or three **(C,D)** independent experiments.

Cells were loaded with 2 μM Fluo-4 (Invitrogen, Waltham, MA, USA) for 20 min at 4°C and 10 min at 37°C, washed once with FACS buffer and resuspended in PBS supplemented with 0.9 mM CaCl_2_ and 0.49 mM MgCl_2_ (Invitrogen, Waltham, MA, USA) and analyzed by flow cytometry (BD FACS-Canto). An infrared lamp was used to maintain a constant sample temperature of 37°C. This was achieved by placing the probe of a digital thermometer in a separate FACS tube filled with PBS in close proximity of the analyzing FACS tube (Figure 1A). After baseline measurement for the indicated times, 1 mM ATP, 1 mM NAD^+^ or 1 mM NAD^+^ + 2 mM dithiothreitol (DTT, Invitrogen, Waltham, MA, USA) was added.

### Pore Formation Assay

Cells were resuspended in PBS supplemented with 0.9 mM CaCl_2_ and 0.49 mM MgCl_2_ (Invitrogen, Waltham, MA, USA) and DAPI was added to a final concentration of 1.5 μM. Cells were analyzed by flow cytometry (BD FACS-Canto) using an infrared lamp to maintain a constant sample temperature of 37°C, as described above. After baseline measurement for the indicated times, 1 mM ATP, 1 mM NAD^+^ or 1 mM NAD^+^ + 2 mM DTT was added.

### LDH Assay

LDH release from mixed glial cells was measured after incubation of cells for 24 h by using the Cytotoxicity Detection Kit (Roche, Basel, Switzerland) in order to estimate the frequency of dead cells after NAD/ATP treatment. The assay was used according to manufacturer’s instructions.

### Etheno-ADP-Ribosylation Assay

Cultured glial cells were incubated for 20 min at 4°C with 100 μM etheno-nicotinamide adenine dinucleotide (etheno-NAD^+^, Sigma-Aldrich, St. Louis, MO, USA) in the presence or absence of 2 mM DTT. Etheno-NAD^+^ was removed by washing cells twice with FACS buffer. Etheno-ADP-ribose bound to cell surface proteins was detected using fluorochrome-conjugated etheno-adenosine-specific monoclonal antibody 1G4, as described previously (Krebs et al., 2003; Rissiek et al., 2017). Cells were washed twice with FACS buffer and analyzed by flow cytometry. Cells that were not treated with etheno-NAD^+^ were stained with 1G4 and used as control.

### HEK Cell Transfection

For transfection experiments pCMVSport6.1 plasmids containing mouse P2X7a or P2X7k were used. Expression constructs were transfected into human embryonic kidney (HEK) cells using jetPEI transfection reagent (Polysciences Europe, Hirschberg an der Bergstraße, Germany). Transfected cells were FACS sorted every 3–4 days for high P2X7 expression in order to generate stably transfected HEK cells. These cells were then directly used in experiments or co-transfected with pCMVSport6 encoding for ARTC2.1 in order to evaluate the impact of ADP-ribosylation.

### P2X7 Splice Variant Typing

RNA was extracted from FACS sorted murine immune cells (astrocytes and microglia from mixed glial cell cultures, peritoneal macrophages, and spleen CD4 T cells) using RNeasy^®^ Plus Mini Kit (Qiagen, Venlo, Netherlands) followed by cDNA synthesis using the Maxima First Strand cDNA Synthesis Kit (Thermo Fisher Scientific, Waltham, MA, USA) as recommended by the respective supplier. P2X7 splice variant determination was performed by polymerase chain reaction (PCR) using forward primers specific to exon 1 of P2X7k (5′- gcccgtgagccacttatgc -3′) and P2X7a (5′- cacatgatcgtcttttcctac -3′) and a common reverse primer, which binds to the exon 5 (5′- ccttgtcttgtcatatggaac -3′) of both splice variants. The amplification conditions were 30 cycles of 94°C for 30 s, 55°C for 30 s, followed by 72°C for 30 s, and the final elongation step at 72°C for 6 min. Due to the low expression level of P2X7 on astrocytes, the number of cycles was elevated to 40 to increase the yield of astrocyte-specific P2X7 transcripts. P2X7a (~380 bp) or P2X7k (~460 bp) were separated in agarose gel electrophoresis (1.5% agarose).

### Software and Statistics

Analysis of flow cytometric data was performed using FlowJo (Treestar). Statistical analyses were performed using Prism 8 software. Two groups were compared by using student’s *t*-test and data is presented as mean ± SD.

## Results

### Astrocytes and Microglia Show No Signs of P2X7 Activation in Response to NAD^+^

We recently identified several target proteins of ARTC2.1 on microglia, including P2X7 (Rissiek et al., 2017). Yet, it is unclear whether the ADP-ribosylation of P2X7 on microglia also induces P2X7 activation. Interestingly, a recent study suggests that astrocytes react to NAD^+^ in an ARTC2/P2X7-dependent fashion, ultimately resulting in astrocyte cell death (Wang et al., 2012). In order to evaluate the impact of P2X7 ADP-ribosylation on microglia and astrocytes, we set up mixed glial cell cultures from neonatal Balb/c mice consisting mainly of astrocytes and microglia. First, we analyzed the impact of NAD^+^ on the immediate effects of P2X7 activation, such as calcium influx and pore formation, using real-time flow cytometry. To distinguish astrocytes and microglia in mixed glial cultures, we used anti-GLAST (astrocytes) and anti-CD11b (microglia) fluorochrome-conjugated antibodies. After 2 min of measuring the baseline signal at 37°C in the absence of an external stimulus, we added either ATP (1 mM) or NAD^+^ (1 mM) to the sample and continued measuring for 6–8 min. Treatment with ATP induced a rapid influx of calcium into astrocytes and microglia and pore formation, as evidenced by uptake of the DNA staining dye DAPI. In contrast, treatment with NAD^+^ neither induced calcium influx nor pore formation in astrocytes and microglia (Figure 1B). Of note, DAPI appeared to diffuse into astrocytes over time. However, the addition of NAD^+^ did not enhance DAPI uptake into astrocytes. We repeated our experimental setup with primary microglia from the brain of adult mice. Again, we observed calcium influx and DAPI uptake in response to ATP but not to NAD^+^ stimulation. As a positive control we used CD4^+^ T cells from spleen, which are known to be able to induce P2X7 activation *via* ADP-ribosylation (Adriouch et al., 2008). Here, we detected calcium influx and DAPI uptake in response to ATP or NAD^+^ stimulation (Figure 1B).

We next compared the capability of NAD^+^ and ATP to induce cell death in mixed glial cultures. We incubated mixed glial cells with rising concentrations of NAD^+^ (1–10 mM) or ATP (5 mM) for 24 h and measured cell death by the release of lactate dehydrogenase (LDH). Since ARTC2 isoforms ARTC2.1 and ARTC2.2 are differentially expressed among inbred mouse strains (Koch-Nolte et al., 1999) we analyzed mixed glial cultures from Balb/c (ARTC2.1^+^/ARTC2.2^+^), C57BL/6 (ARTC2.1^−^/ARTC2.2^+^), NZW (ARTC2.1^+^/ARTC2.2^−^), and the outbred strain ICR. Interestingly, treatment with up to 10 mM NAD^+^ did not induce detectable cell death in mixed glial cultures from all analyzed mouse strains. In contrast, 5 mM ATP induced cell death of 40–70% of mixed glial cells from all analyzed strains (Figure 1C).

Since a lack of response to treatment with NAD^+^ could be a result of low or absent ecto-ART activity, we next stimulated mixed glial cells from Balb/c mice with LPS and U0126 in order to induce ARTC2.1 expression (Hong et al., 2007, 2009a; Rissiek et al., 2017). To measure ecto-ART activity we incubated LPS/U0126 stimulated and unstimulated mixed glial cells with etheno-NAD^+^ and the reducing agent dithiothreitol (DTT) in order to enhance ARTC2.1 activity (Hara et al., 2000). Incorporation of etheno-ADP-ribose into cell surface proteins was detected with the etheno-ADP-ribose-specific monoclonal antibody 1G4 (Young and Santella, 1988; Krebs et al., 2003). Indeed, treatment with LPS/U0126 markedly increased the ecto-ART activity of both, astrocytes and microglia (Figure 1D). We next tested the impact of NAD^+^/DTT treatment on LPS/U0126 stimulated mixed glial cells using the calcium flux and pore formation assays. Again, we did not detect any notable response to NAD^+^/DTT treatment, whereas ATP stimulation-induced calcium influx and pore formation, as shown before (Figure 1E). Finally, we evaluated the impact of NAD^+^/DTT on the induction of cell death in LPS/U0126 stimulated mixed glial cultures. Of note, neither NAD^+^ alone nor NAD^+^/DTT did induce LDH release from Balb/c mixed glial cultures. In contrast, ATP stimulation induced cell death in a substantial fraction of LPS/U01267 stimulated mixed glial cells, even stronger when compared to unstimulated control (Figure 1F). In summary, ATP but not NAD^+^ induced activation of P2X7 on astrocytes and microglia, and this did not change after LPS/U0126-induced increase of cell surface ART-activity.

### ADP-Ribosylation of P2X7 Can Be Detected on Microglia but Not on Astrocytes

**Figure 2:**
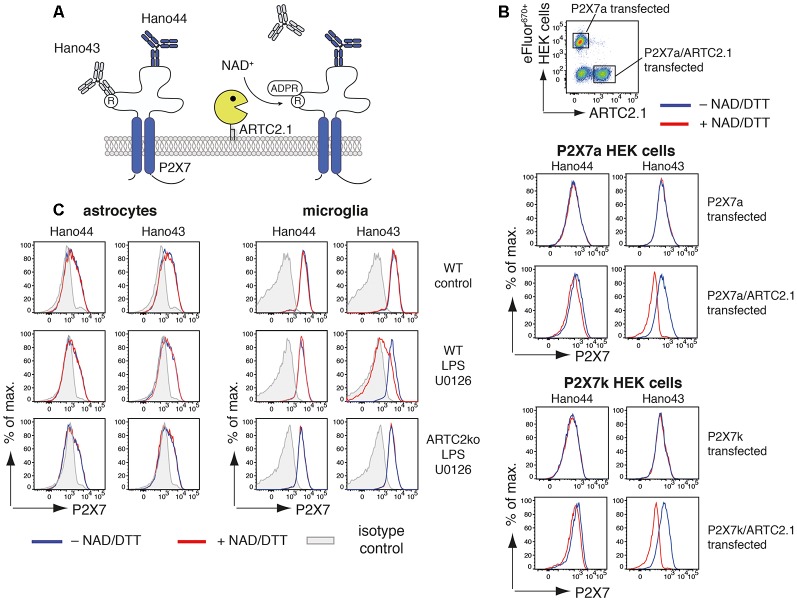
Antibody-aided detection of P2X7-ADP-ribosylation. **(A)** Schematic of the antibody-aided ADP-ribosylation assay. Hano43 and Hano44 are two P2X7-specific mAbs that bind distinct epitopes on P2X7. Binding of Hano43 but not of Hano44 is blocked by ADP-ribosylation of P2X7. **(B)** HEK cells, stably transfected with mouse P2X7a or P2X7k splice variants, were transiently co-transfected with mouse ARTC2.1. HEK cells only expressing P2X7 were labeled with eFluor^670^ and mixed with unlabeled P2X7/ARTC2.1 co-transfected HEK cells. Mixed cells were incubated with or without NAD^+^/DTT for 20 min at 4°C and binding of Hano43 and Hano44 was measured by flow cytometry. Gating was performed on eFluor^+^ P2X7-transfected or eFluor^−^ ARTC2.1/P2X7 co-transfected HEK cells. **(C)** Astrocytes and microglia from mixed glial cells of Balb/c WT or Balb/c ARTC2ko mice were stimulated or not with LPS/U0126 overnight. ADP-ribosylation of P2X7 after incubation with or without NAD^+^/DTT was measured by binding of Hano43/Hano44 in comparison to isotype control. Gating was performed on CD11b^+^GLAST^−^ (microglia) and CD11b^−^GLAST^+^ (astrocytes) cells. Data represent results from two independent experiments.

Since treatment with NAD^+^ did not induce any detectable activation of P2X7 we analyzed the ability of ARTC2.1 to ADP-ribosylate P2X7 on astrocytes and microglia. For this, we utilized a pair of P2X7-specific monoclonal antibodies that are differentially affected by ADP-ribosylation of P2X7: binding of Hano43 to P2X7 is inhibited by ADP-ribosylation of P2X7 whereas Hano44 binds both, ADP-ribosylated and unmodified P2X7 (Figure 2A). HEK cells, stably transfected with either P2X7a or P2X7k, were labeled with eFluor^670^ and mixed with HEK cells that were additionally transiently transfected with ARTC2.1 (Figure 2B). Treatment of ARTC2.1 co-transfected HEK cells with NAD^+^/DTT resulted in a strongly reduced binding of Hano43 but not of Hano44. In contrast, staining with Hano43 was not reduced after the treatment of HEK cells that were only transfected with P2X7a or P2X7k with NAD^+^/DTT. Of note, Hano44 binding was comparable among P2X7-transfected and P2X7/ARTC2.1 co-transfected HEK cells, regardless of NAD^+^/DTT treatment.

We next applied this tool for measuring ADP-ribosylation of P2X7 in mixed glial cell cultures that had been stimulated for 24 h with or without LPS/U0126 (Figure 2C). For microglia but not for astrocytes treatment with NAD^+^/DTT resulted in a reduced binding of Hano43. Reduced binding of Hano43 was not observed for microglia obtained from ARTC2ko mice, indicating that this effect indeed is mediated by ARTC2.1-catalyzed ADP-ribosylation of P2X7.

### Microglia and Astrocytes Express the P2X7a Variant Which Is Insensitive to ADP-Ribosylation

**Figure 3:**
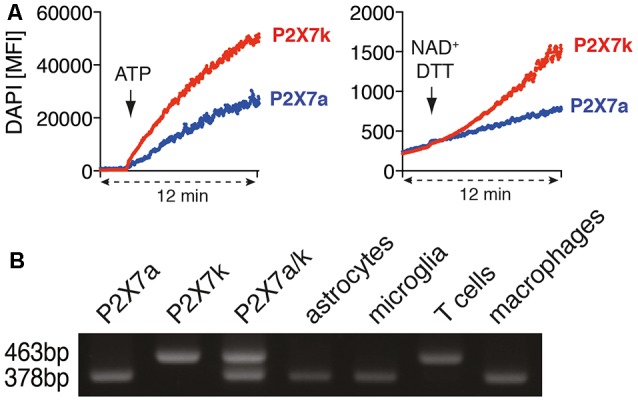
Astrocytes and microglia express the ADP-ribosylation insensitive P2X7a splice variant. **(A)** P2X7a transfected HEK cells were labeled with eFluor670 and mixed with unlabeled P2X7k transfected HEK cells in PBS supplemented with calcium and DAPI. DAPI uptake was measured for 2 min, then ATP (1 mM) or NAD+/DTT was added and measuring continued for 10 min. **(B)** cDNA from astrocytes, microglia, spleen T cells and peritoneal macrophages was used for P2X7 splice variant-specific PCR. Expression plasmids for P2X7a (PCR product: 378bp) and P2X7k (PCR product: 463 bp) or a mixture of P2X7a/k was used as a positive control for the splice variant-specific PCR. The shown data represent results from two independent experiments.

T cells and macrophages express different P2X7 splice variants (Nicke et al., 2009). With our antibody-based ADP-ribosylation assay of P2X7 on HEK cells we could demonstrate that both splice variants can be ADP-ribosylated by ARTC2.1. It has been reported that ADP-ribosylation can trigger the gating of P2X7k but not of P2X7a (Schwarz et al., 2012). Indeed, treatment of P2X7k/ARTC2.1-transfected HEK cells but not of P2X7a/ARTC2.1 transfected HEK with NAD^+^/DTT induced uptake of DAPI (Figure 3A). In contrast, treatment with ATP induced DAPI uptake in both, P2X7k/ARTC2.1- and P2X7a/ARTC2.1-transfected HEK cells.

To determine whether astrocytes or microglia express the P2X7a and/or P2X7k splice variant, we performed a splice variant-specific PCR analysis. As controls we used P2X7a- or P2X7k- expression plasmids and cDNA from peritoneal macrophages, known to express primarily P2X7a, and spleen CD4^+^ T cells, known to express primarily P2X7k (Schwarz et al., 2012). The results show that both, astrocytes and microglia, predominantly express the ADP-ribosylation-insensitive P2X7a variant (Figure 3B). This provides a possible explanation for the apparent resistance of astrocytes and microglia towards NAD-induced cell death along the ARTC2/P2X7 axis.

## Discussion

In this study, we evaluated astrocytes and microglia for their sensitivity towards NAD^+^-mediated activation of the ARTC2/P2X7 axis. We found that ATP but not NAD^+^ induced activation of P2X7 in astrocytes and microglia, even if cell surface ecto-ART activity on both cell types was increased by treatment with LPS/U0126 for 24 h. Consistently, astrocytes and microglia in mixed glial cultures were resistant to NAD^+^-induced cell death (NICD) but not to ATP-induced cell death. For astrocytes, a possible explanation for NICD resistance is insufficient ADP-ribosylation of P2X7. The capacity of astrocytes to ADP-ribosylate cell surface proteins is lower than that of microglia (see Figure 1C). Moreover, the cell surface density of P2X7 is also lower on astrocytes than on microglia (see Figure 2C).

Our antibody-based detection system for the ADP-ribosylation of P2X7 is not unique to P2X7. Loss of antibody-binding to ADP-ribosylated proteins has also been described for other ARTC2 target proteins: ADP-ribosylation of CD25 leads to a loss of binging of the clone 7D4 but not of PC61 (Teege et al., 2015). For CD8β, ADP-ribosylation diminishes binding of clones YTS156.7.7 and 53-5.8 but has no influence on the binding of 53-6.7 and H35-17.2 (Lischke et al., 2013). For LFA-1, ADP-ribosylation decreased the binding of mAbs 2D7 and C71/16 but not of mAb M17/4 (Nemoto et al., 1996)). This suggests that pairs of monoclonal antibodies that are affected/unaffected by ADP-ribosylation could be used as a non-radioactive alternative approach to estimate specific target ADP-ribosylation.

Another possible explanation for the lack of detectable P2X7 ADP-ribosylation on astrocytes could be the removal of the ADP-ribose groups by other cell surface enzymes (Zolkiewska and Moss, 1995; Nemoto et al., 1996; Laing et al., 2011). One possible candidate is ectonucleotide pyrophosphatase/phosphodiesterase 1 (ENPP1), which can hydrolyze AMP from protein-attached ADP-ribose groups, yielding proteins modified with ribose-5’-phosphate (Palazzo et al., 2016). Whether ENPP1 is expressed by astrocytes or microglia and is capable of partially reversing cell surface protein ADP-ribosylation on these cells still needs to be investigated.

One important finding of this study is the identification of P2X7a as a predominant splice variant in astrocytes and microglia. Since P2X7a is not gated by ADP-ribosylation (Schwarz et al., 2012), this could account for the resistance of astrocytes and microglia towards NICD. P2X7a and P2X7k differ in their cytosolic N-terminus as well as and in most of the first transmembrane domain (Nicke et al., 2009). Interestingly, by mutating the arginine at position 276 into lysine (R276K), P2X7a can be made sensitive to ADP-ribosylation (Schwarz et al., 2009, 2012). This gives room for speculation that also other modifications e.g., binding of small molecules or other proteins could render P2X7a sensitive to ADP-ribosylation.

Our results are in contrast to those of a previously published study that implicated NAD^+^ as an inducer of astrocyte cell death in an ARTC2/P2X7 dependent fashion (Wang et al., 2012). However, Wang et al. neither analyzed whether astrocytes exhibit ecto-ART activity nor whether P2X7 on astrocytes is subject to ADP-ribosylation. Therefore, probably the NAD^+^ mediated impact on astrocyte vitality could be triggered by ARTC2/P2X7-independent signaling pathways. Indeed, NAD^+^ can also serve as ligand for other receptors, such as the metabotropic P2Y1 receptor (Mutafova-Yambolieva et al., 2007; Hwang et al., 2012). P2Y1 is expressed by astrocytes (Bowser and Khakh, 2004), however, to date it has not been reported that activation of P2Y1 by extracellular NAD^+^ or its other ligand ADP can induce cell death in astrocytes. Further, it is conceivable that metabolites of NAD^+^ rather than NAD^+^ itself trigger astrocyte cell death. Astrocytes reportedly express the NAD^+^-hydrolyzing ecto-enzyme CD38 (Yamada et al., 1997) that generates extracellular cyclic ADP-ribose and ADP-ribose. Therefore, future studies will show whether these NAD^+^ metabolites influence astrocyte vitality.

## Data Availability Statement

The datasets generated for this study are available on request to the corresponding author.

## Ethics Statement

The animal study was reviewed and approved by Hamburger Behörde für Gesundheit und Verbraucherschutz, Veterinärwesen/Lebensmittelsicherheit.

## Author Contributions

BR, JS and MC performed the experiments with mixed glial cultures. BR and YD performed the experiments involving transfected HEK cells. SB perform P2X7 splice variant typing. SM, FK-N and TM supervised the experiments and assisted with data interpretation and manuscript preparation. BR assembled the figures and wrote the manuscript, which has been reviewed by all authors.

## Conflict of Interest

FK-N receives royalties from sales of antibodies developed in the lab *via* MediGate GmbH, a 100% subsidiary of the University Medical Center, Hamburg. The remaining authors declare that the research was conducted in the absence of any commercial or financial relationships that could be construed as a potential conflict of interest.
